# Driving risk cognition of passengers in highly automated driving based on the prefrontal cortex activity via fNIRS

**DOI:** 10.1038/s41598-023-41549-9

**Published:** 2023-09-22

**Authors:** Hong Wang, Xiaofei Zhang, Jun Li, Bowen Li, Xiaorong Gao, Zhenmao Hao, Junwen Fu, Ziyuan Zhou, Mohamed Atia

**Affiliations:** 1 School of Vehicle and Mobility, Tsinghua University, Beijing, 100084 China; 2 School of Medicine, Tsinghua University, Beijing, 100084 China; 3 School of Computer Science, Carleton University, Ottawa, ON K1S5B6 Canada; 4 Department of Systems and Computer Engineering, Carleton University, Ottawa, ON K1S5B6 Canada

**Keywords:** Mechanical engineering, Electrical and electronic engineering

## Abstract

For high-level automated vehicles, the human being acts as the passenger instead of the driver and does not need to operate vehicles, it makes the brain–computer interface system of high-level automated vehicles depend on the brain state of passengers rather than that of drivers. Particularly when confronting challenging driving situations, how to implement the mental states of passengers into safe driving is a vital choice in the future. Quantifying the cognition of the driving risk of the passenger is a basic step in achieving this goal. In this paper, the passengers’ mental activities in low-risk episode and high-risk episode were compared, the influences on passengers’ mental activities caused by driving scenario risk was first explored via fNIRS. The results showed that the mental activities of passengers caused by driving scenario risk in the Brodmann area 10 are very active, which was verified by examining the real-driving data collected in corresponding challenging experiments, and there is a positive correlation between the cerebral oxygen and the driving risk field. This initial finding provides a possible solution to design a human-centred intelligent system to promise safe driving for high-level automated vehicles using passengers’ driving risk cognition.

## Introduction

With the spread adoption of artificial intelligence (AI), the great challenges confronted by intelligent safety have gained increasing attention, and become the biggest obstruction to the mass production of high-level automated vehicles nowadays. The chief important reason for these accidents happening in recent years is that there are functional deficiencies in robustness, generalization and so on, particularly perception algorithm deficiency. Such functional deficiencies may lead to the safety of the intended functionality (SOTIF)^1,2^, which means the absence of unreasonable risk due to hazards resulting from functional insufficiencies of the intended functionality or reasonably foreseeable misuse by person^1^. For example One high-level automated vehicle hit a white overturned truck as a result of the white truck is mistakenly identified as a white cloud. In another case, an automated vehicle hit one 40-year-old woman who was crossing the street during night, because the decision-making algorithm of the vehicle ignored people crossing the road illegally^3^.

The unexplained black box properties of AI algorithms make it challenging to overcome its functional deficiencies by improving AI algorithms. It is important to overcome its functional deficiencies using the brain–computer interface technology by studying the relationship between passengers’ mental activity and driving scenario risk and implementing the cognition of the most advanced senor-human being into safe driving. The characteristics of functional near-infrared spectroscopy (fNIRS) technology, such as non-invasiveness, safety, and low-cost characteristics, make fNIRS own potential as an ideal candidate for monitoring the brain activity^4–12^. Some researches have been done for looking into the mental activity of drivers on driving simulators or highways for different driving tasks^13–19^. For improving traffic safety, the current mental workload of drivers has also been investigated^20^, the data from four regions of interest, i.e, the left anterior dorsolateral prefrontal cortex (DLPFC), the left posterior DLPFC, the right anterior DLPFC and the right posterior DLPFC were analyzed. The following three points were obtained: (1) All four regions of interest showed significant differences between conditions in deoxygenated hemoglobin, and it indicates higher activity during higher subjective workload ratings during city courses than lower subjective workload ratings during country courses. (2) No region of interest showed significant differences among the conditions in oxygenated hemoglobin. (3) The left anterior DLPFC region is the most sensitive to mental workload changes, and the right middle frontal gyrus might be a suitable region for the application of powerful small-area brain–computer interfaces. The relationship between cortical activity and the levels of smartphone distraction was explored^14^, and it has found that the prefrontal cortical activities of drivers are sensitive to mental workload changes and the levels of smartphone distraction. The cognitive processes related to driving were explored, and it was noted that additional neural resources are needed in the prefrontal cortex during high-speed driving conditions compared to the lower-speed cases during dual task driving^8^. A study on the relationship between the prefrontal cortex activation and the changes in mental workload during simulated driving showed that the increases in the subjective ratings of mental workload are associated with increases in the concentration of oxygenated hemoglobin in the prefrontal cortex^19^. As stated above, the prefrontal cortices are related to driving tasks and mental workload, thus prefrontal cortices are the regions of interest for our study^8,14,19,20^. Gauvain Huve^21^ presented a brain-computer interface system, and this system may analyze the brain activity of a user in real time and deduce the current driving mode of the car. This study is important to ascertain if a driver is fit to drive at any given time in case the auto-pilot fails, but the results^21^ are not applicable to high-level automated vehicles. In high-level automated vehicles, the human being acts as the passenger instead of the driver and does not need to operate the vehicle, therefore, it is very meaningful to improve SOTIF using the brain-computer interface based on the cortical activities of passengers caused by driving scenario risk.

The choice of data features is also crucial for result analysis, there are four indexes, the changes in the concentration of oxygenated hemoglobin $\triangle HbO$, deoxygenated hemoglobin $\triangle HbR$, cerebral blood volume $\triangle CBV$ and cerebral oxygen exchange $\triangle COE$^22–24^. Based on these four indexes, the time, frequency and wavelet features can be obtained. WeiTa Chen^24^ used some features from those four indexes to classify healthy subjects, chronic migraine subjects and medication-overuse headache subjects using machine learning. Raul Fernandez Rojas^25^ studied the classification results of seven machine learning algorithms for identifying a biomarker of human pain using fNIRS, and different machine learning algorithms achieved different classification accuracy based on different features. The Gaussian support vector machines presented the highest accuracy (94.17%) using only 25 features to identify the four types of pain in a database, and this result indicated that the choice of data features is crucial.

$\triangle COE$ is an effective index that can indicate activity^26–28^, dictionary learning is a parametric low-dimensional representation learning method that can reconstruct high-dimensional input vectors in an unsupervised manner, and it is also a typical feature extraction method of machine learning. Compared with regular features, dictionary learning owns learning characteristics, and can find the optimal characteristic to reconstruct high-dimensional input vectors. Human-centred intelligent system is useful to promise safe driving for high-level automated vehicles. It is a possible and novelty solution to design a human-centred intelligent system using passengers’ driving risk cognition. In order to achieve this goal, the influences on mental activity caused on driving scenarios risks based on blood oxygen monitoring by fNIRS technology was studied. Firstly, a signal acquisition system and challenging driving scenarios are designed by hardware-in-loop equipment and Virtual Test Drive software, an experiment which contains different driving scenarios was conducted, 20 participants completed this experiment, and a blood oxygen monitoring named OctaMon+ that owns eight channels was used, in this study. Secondly, a K-SVD (it does K iterations of singular value decomposition ) dictionary^29^ learning and mean value methods were used to extract features from $\triangle COE$ data. T-test^14^ and generalized linear mode (GLM)^5,30–32^ were adopted to analyze the sensitive area to driving scenario risk and the quantification relationship between passengers’ mental activity and driving scenario risk based on those features. Finally, some possible contributions to improve SOTIF based on the conclusion of this paper were introduced.

## Results

It is possible and novelty to improve SOTIF by designing a human-centred intelligent system using passengers’ driving risk cognition. In this paper, driving scenario is divided into a low-risk episode and a high-risk episode based on an objective risk evaluation indicator, and passengers’ mental activities in low-risk episode and high-risk episode were compared. This experiment was proceeded on a driving simulator, and 20 participants completed this experiment. The data of four challenging driving scenarios were analyzed: lead vehicle autonomous emergency braking in a short distance, lead vehicle cut-in from left lane in a short distance, lead vehicle cut-in from right lane in a short distance and pedestrian crossing road from right. The valid sample numbers of lead vehicle autonomous emergency braking in a short distance, lead vehicle cut-in from left lane in a short distance, lead vehicle cut-in from right lane in a short distance and pedestrian crossing road from right are 267, 531, 480 and 632. The findings were as follows: (1) The mental activities of passengers caused by driving scenario risk in the channel 8 (Brodmann area 10, Left Cerebrum, Frontal Lobe, Inferior Frontal Gyrus, Gray Matter ) were very active, and this initial conclusion was also verified by examining the real-driving data collected in corresponding challenging experiments that were performed in the experimental base in Changsha, China. (2) The mental activity caused lateral risk is stronger than longitudinal risk. (3) There is a positive correlation between the cerebral oxygen exchange and risk field, and this correlation may be modeled as a linear relationship by GLM. This difference of mental activity between low-risk and high-risk episodes may be combined with reinforcement learning to realize passenger-in-loop decision-making. It provides a possible solution to design a human-centred intelligent system to improve SOTIF for high-level automated vehicles using passengers’ driving risk cognition.

## Discussion

**Figure 1:**
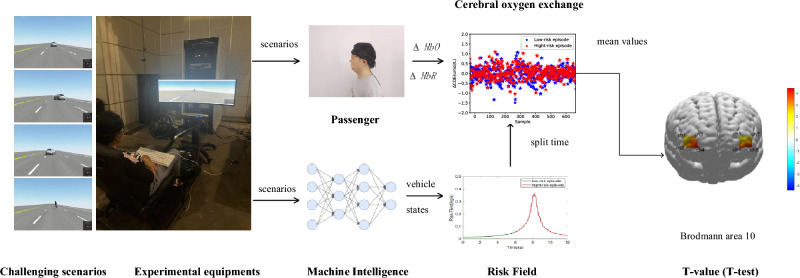
The flow diagram contains cerebral oxygen exchange and risk field data. Each scenario is divided into low-risk and high-risk episodes based on risk field data, the difference of cerebral oxygen exchange between low-risk and high-risk episode is analyzed using T-value, which may indicate the mental activity differences caused by risk field.

In this paper, firstly, a signal acquisition system and challenging driving scenarios were designed by hardware-in-loop equipment and Virtual Test Drive software; Secondly, vehicle states, which may be used to build risk field, and $\triangle HbO$ and $\triangle HbR$ of passengers, were collected by the signal acquisition system. Thirdly, cerebral oxygen exchange was divided into low-risk and high-risk episodes based on a risk field; Finally, the mean values about risk fields in low-risk and high-risk episodes were compared, the differences of passengers’ mental activities between low-risk and high-risk episodes were analyzed using T-value of T-test, and the quantification relationship between passengers’ mental activity and driving scenario risk was explored. The flow diagram is showed in Fig. 1.

### Risk analysis

Risk field is a safety indicator, it may quantify driving scenario risk, it involves relative longitudinal distance, relative speed, and those information is related to time to collision. To analyze the risk field differences between low-risk and high-risk episodes, the mean values of the risk field were calculated, and the detailed results are shown in Table 1.

**Table 1:** Difference of risk field between low-risk and high-risk episodes.

Event	$M_L$	$M_H$	$D_{LH}$	*p*	*t*
Lead vehicle autonomous emergency braking in a short distance scenario	0.010	0.128	0.118	$<0.01$	20
Lead vehicle cut-in from left lane in a short distance scenario	0.013	0.124	0.111	$<0.01$	15
Lead vehicle cut-in from right lane in a short distance scenario	0.016	0.162	0.146	$<0.01$	10
Pedestrian crossing road from right scenario	0.022	0.116	0.094	$<0.01$	6

(1) $M_L$ indicates the mean values of the risk field in the low-risk episode; (2) $M_H$ indicates the mean values of the risk field in the high-risk episode; (3) $D_{LH}$ indicates the difference of risk field between low-risk and high-risk episodes; (4) *p* indicates the probability of T-test; (5) *t* indicates the time of episode.

The *p* values of those four challenging scenarios are smaller than 0.1, which indicates that there are significant difference about risk field between low-risk episode and high-risk episode. The mean value of high-risk episode is higher than that of the low-risk episode. Compared with lead vehicle autonomous emergency braking in a short distance, lead vehicle cut-in from left lane in a short distance, lead vehicle cut-in from right lane in a short distance scenarios, the risk field change trend of pedestrian crossing road from right scenario is most fastest (it completed the change from minimum value to maximum value in 6 s).

### Mental activity analysis

**Figure 2:**
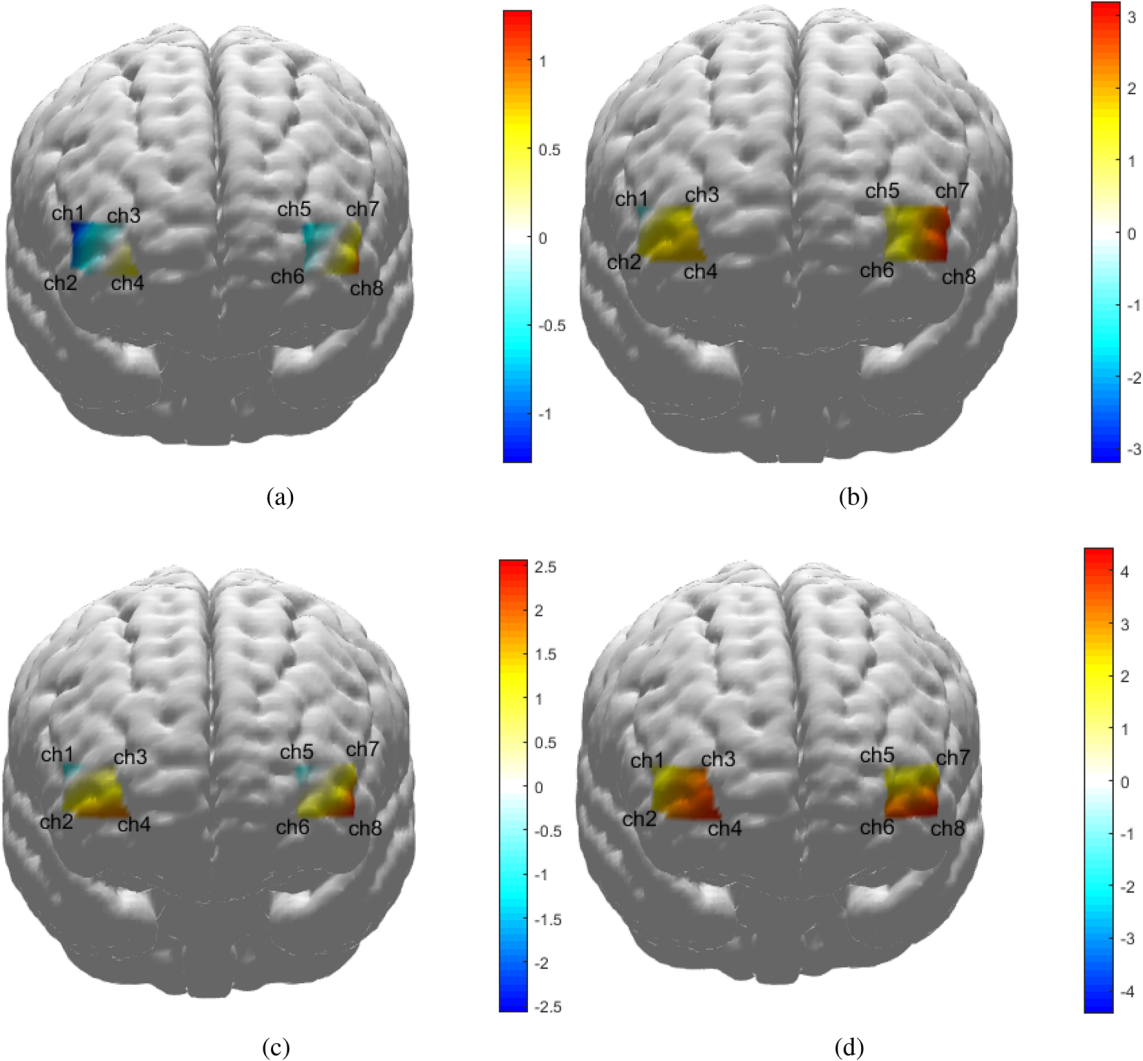
The T-values of $\triangle COE$ based on mean value features between low-risk episode and high-risk episode in driving scenarios of lead vehicle autonomous emergency braking in a short distance, lead vehicle cut-in from left lane in a short distance, lead vehicle cut-in from right lane in a short distance and pedestrian crossing road from right. are shown in (**a**–**d**).

In this study, $\triangle COE$ was utilized to indicate mental activity, the differences of $\triangle COE$ between low-risk and high-risk episodes were compared using the T-value. Mean method and K-SVD dictionary were used to extract feature from raw data. The differences in the T-value based on mean value features between low-risk and high-risk episodes are shown in Fig. 2, and the T-values based on mean value features and the T-value based on K-SVD dictionary features are shown in Table 2. From the T-value result based on mean value features, the following findings can be derived: (1) The T-values of lead vehicle autonomous emergency braking in a short distance, lead vehicle cut-in from left lane in a short distance, lead vehicle cut-in from right lane in a short distance and pedestrian crossing road from right in the channel 8 are maximum, compared with other channels. The T-value for lead vehicle autonomous emergency braking in a short distance is 1.199, the T-value for lead vehicle cut-in from left lane in a short distance is 3.205, the T-value for lead vehicle cut-in from right lane in a short distance is 2.572, the T-value for pedestrian crossing road from right is 4.439. This indicates that the mental activities of passengers caused by driving scenario risk in the Brodmann area 10 is very active. (2) The T-values in channel 8 of lead vehicle cut-in from left lane in a short distance, lead vehicle cut-in from right lane in a short distance and pedestrian crossing road from right all bigger than the T-value in channel 8 of lead vehicle autonomous emergency braking in a short distance. This means that the mental activity caused lateral risk may be stronger than longitudinal risk. (3) Compared with other three scenarios, the mental activity in the channel 8 of pedestrian crossing road from the right scenario is the most obvious ( the T-values in channel 8 is maximum ), it may be related to the risk field change trend (the risk field change trend of pedestrian crossing road from the right scenario is most fastest).

K-SVD dictionary learning is a typical feature extraction method of machine learning, it is often used in image processing field, so we try to analyze the results based on K-SVD dictionary features. The K-SVD dictionary features do not stand for any contents, so the symbol of T-value based on K-SVD dictionary features has no significance. However, the absolute T-value may indicate the different degree of $\triangle COE$ between low-risk and high-risk episodes. The absolute T-values in channel 8 of lead vehicle cut-in from left lane in a short distance, lead vehicle cut-in from right lane in a short distance and lead vehicle autonomous emergency braking in a short distance are maximum, compared with other channels. Those results were obtained based on python 3.11.3 and numpy 1.24.3. However, the absolute T-values in channel 4 of pedestrian crossing road from right scenario is maximum. The reason is that K-SVD dictionary learning depends on singular value decomposition.The essence of singular value decomposition result in there are a performance in which the signs of K-SVD dictionary features are opposite in two calculations for same data sometimes. So the K-SVD dictionary features are not unsuited for analysing the differences of $\triangle COE$ between low-risk and high-risk episodes based on T-test.

**Table 2:** The T-test results of $\triangle COE$ between low-risk and high-risk episodes.

	Scenario	Channel 1	Channel 2	Channel 3	Channel 4	Channel 5	Channel 6	Channel 7	Channel8
Mean Value Feature	SAEB	$-1.284$	$-0.817$	$-0.181$	0.745	$-0.844$	$-0.145$	0.426	**1.199**
	LCI	$-1.181$	1.610	1.529	1.884	0.797	0.998	2.690	**3.205**
	RCI	$-1.120$	1.287	0.451	1.907	$-1.002$	0.888	1.102	**2.572**
	RPCR	1.139	2.343	3.052	4.043	1.075	3.423	1.755	**4.439**
K-SVD Dictionary Feature	SAEB	1.007	$-1.941$	$-1.563$	$-0.335$	$-1.382$	1.406	$-1.879$	−**4.214**
	LCI	0.665	1.934	0.306	1.514	$-0.717$	$-0.202$	$-2.828$	−**3.609**
	RCI	0.055	-0.532	0.474	1.832	-1.003	-0.595	-2.088	**-2.524**
	RPCR	−0.160	2.394	−3.057	4.052	0.388	3.435	1.769	0.552

(1) SAEB stands for autonomous emergency braking in short-distance scenario; (2) LCI stands for the vehicle cut in from the left lane scenario; (3) RCI stands for the vehicle cut in from the right lane scenario; (4) RPCR stands for the pedestrian crossing road from the right scenario.

### The influences on mental activity caused by sex and driving experience

**Figure 3:**
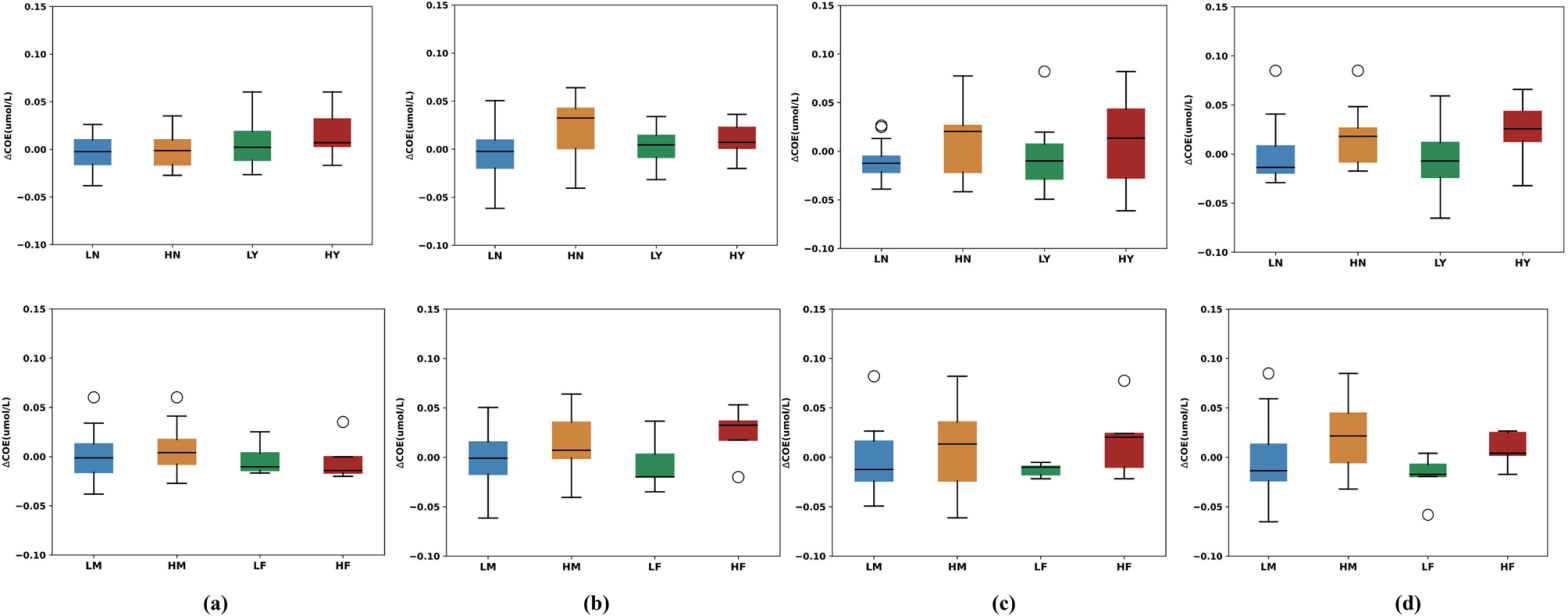
The boxplots of cerebral oxygen exchange in channel 8 about the driving experience and sex. The boxplots of autonomous emergency braking in a short distance, lead vehicle cut-in from the left lane in a short distance, lead vehicle cut-in from the right lane in a short distance, and pedestrian crossing road from right scenarios are shown in (**a–d**). LN stands for the low-risk episode data about those participants who do not have valid driving experience; HN stands for the high-risk episode data about those participants who do not have valid driving experience; LY stands for the low-risk episode data about those participants who have valid driving experience; HY stands for the high-risk episode data about those participants who have valid driving experience; LM stands for the low-risk episode data about those male participants; HM stands for the high-risk episode data about male participants; LF stands for the low-risk episode data about female participants; HF stands for the high-risk episode data about female participants.

There are 20 participants (mean age standard deviation: 29.45 ± 7.5497; range: 21–46 years), which consist of five females and 15 males, and seven participants have valid driving experience. The boxplots of $\triangle COE$ in channel 8 about driving experience and sex are shown in Fig. 3. The following findings can be derived from Fig. 3. (1) The differences in $\triangle COE$ between low-risk and high-risk episodes in lead vehicle cut-in from the left lane in a short distance, lead vehicle cut-in from the right lane in a short distance, and pedestrian crossing road from right scenarios are more obvious, compared with lead vehicle autonomous emergency braking in a short distance scenario. Besides, the *p* values of autonomous emergency braking in a short-distance scenario, lead vehicle cut-in from left lane in a short distance, lead vehicle cut-in from right lane in a short distance, and pedestrian crossing road from right in channel 8 are 0.232, 0.001, 0.10 and 0.00001 respectively. The value of autonomous emergency braking in short-distance scenarios is greater than the values of three other scenarios. Besides, the values of three other scenarios all are smaller than 0.1, which means that there are significant differences between low-risk and high-risk episodes. So the boxplot result is consistent with the results about values of the t-test based on mean value features, and it indicates that the mental activity caused by lateral risk is stronger than longitudinal risk. (2) For lead vehicle autonomous emergency braking in a short-distance scenario, driving experience may influence the difference of between low-risk and high-risk episodes. This indicates that driving experience makes people more sensitive to longitudinal risks. (3) Similarly, it may be concluded that male participants are more sensitive to longitudinal risks than female participants.

### Quantified cognition of driving risk of passengers

Based on the above discussion, it may be obtained that the mental activities in the channel 8 caused by the driving risk of pedestrian crossing road from the right scenario is the most strongest. Therefore the relationship between the mental activities in the channel 8 and the risk field of pedestrian crossing road from the right scenario was analyzed by GLM, the results are showed in Table 3, and it indicates that there is a positive correlation between the cerebral oxygen exchange and risk field, and this correlation may be modeled as a linear relationship by GLM.

**Table 3:** Parameter estimation.

Parameter	B	Std error	95% Wald confidence interval		Hypothesis test		
			Lower	Upper	Wald Chi-square	df	Sig
(Intercept)	0.069	0.0013	0.066	0.072	2658.780	1	0.00
(Scale)	0.002^a^	8.9830^−5^	0.002	0.002			

(1) Dependent variable: risk field; ( 2) model: (Intercept).

^a^ Maximum likelihood estimate.

### An experiment based on real driving scenarios

**Table 4:** Detailed information of those seven autonomous corresponding challenging cut in from the right lane scenarios.

Order number	Relative velocity (km/h)	Longitudinal speed of ego vehicle (km/h)	Longitudinal speed of target vehicle (km/h)	Lateral speed of target vehicle (m/s)	Relative distance (m)
1	15	60	45	1	10
2	15	60	45	1	8
3	15	60	45	1	6.5
4	15	60	45	1	5
5	15	70	55	0.6	7
6	25	70	45	0.7	12
7	25	70	45	0.9	11

**Figure 4:**
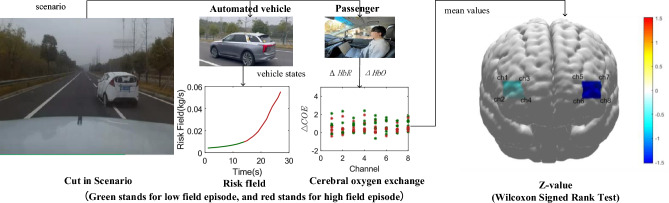
The pieces of equipment which are used in the real driving scenarios experiment contain a high-level automated vehicle and a blood oxygen monitoring device. The Z-values of channels 1, 2, 3, 4, 5, 6, 7 and 8 are $-0.507, -0.169,-0.507, -0.169, -1.521, -1.521, -1.352$, and $-1.521$.

A driving simulator could not reproduce all factors of real driving scenarios, which means there is still exists a difference between simulation and real vehicles. Passenger may be relaxed when a driving simulator is used, so the result of the vehicle cut in from the right lane scenario was verified by the data was collected from real driving scenarios. This experiment was performed in the national intelligent connected vehicle test area in Changsha, China, and a high-level automated vehicle named as E-HS9 from First Automobile Work shop (FAW) was used, and it contains one forward looking monocular camera, one forward millimeter wave radar and four angular millimeter wave radar. Seven autonomous corresponding challenging cut in from the right lane scenarios were designed, and the corresponding cerebral oxygen exchange data were collected, the detailed information is shown in Table 4. The process and results are shown in Fig 4. In this experiment, Wilcoxon Signed Rank Test was used to analyse the difference in cerebral oxygen exchange between low-risk and high-risk episodes of small samples. Firstly, the participant sat at the copilot, and observed in front situations; Secondly, the risk field was built based on vehicle state information, raw data was preprocessed by moving average filter, and the corresponding cerebral oxygen exchange data was divided into low-risk and high-risk episodes based on risk field data; Finally, the difference in $\triangle COE$ between low-risk and high-risk episodes was analyzed using Wilcoxon Signed Rank Test. The absolute Z-values of Wilcoxon Signed Rank Test in channel 5, 6, 8 are maximum, and this shows that there are obvious difference of $\triangle COE$ between low-risk and high-risk episode, compared with other channels.

## Methods

### Participants

**Figure 5:**
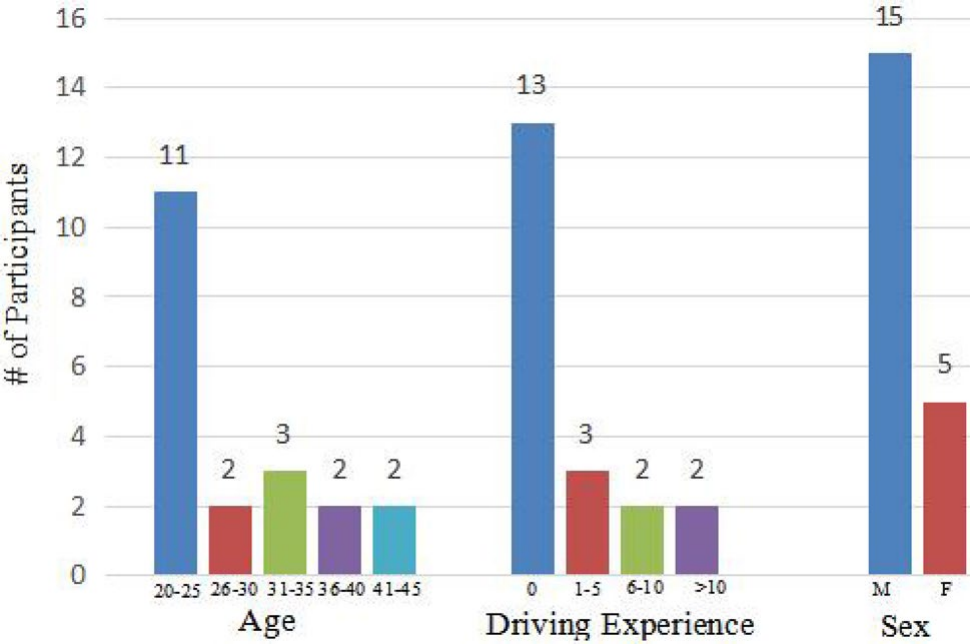
The features of the participants, including age, sex and driving experience (n $=20$).

The features of participants are shown in Fig 5. The detailed information about participants is shown in Supplementary Table 1, the final sample consisted of 20 participants (5 female; mean age standard deviation: 29.45 ± 7.5497; range: 21–46 years). Six participants have valid driving experience and no participant stated disease or predisposition for simulator sickness. Participation was voluntary, informed consent was obtained after the task had been explained, participants were informed that they have an option to end participation in this experiment at any time without any type of penalty. This study complied with the Declaration of Helsinki and was approved by the Institutional Review Board of Tsinghua University, China.

### Experimental paradigm

**Figure 6:**
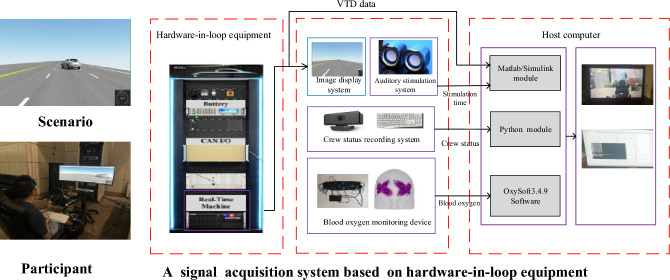
The signal acquisition system based on hardware-in-loop equipment, it can provide scenario information for participants and record vehicle states, the states of participants, and the cerebral oxygen exchange data of passengers.

In this study, the influences on passengers’ mental activities caused by driving scenarios risk was investigated. This experiment was performed in driving simulator. Besides, a signal acquisition system based on hardware-in-loop equipment was designed for this study, it contains Matlab/Simulink module, Python module and OxySoft Software, they can record vehicle states which may be used to build risk field, the state of the participant, and the cerebral oxygen exchange data of passenger, respectively, and it is shown in Fig. 6. The structure of this human factor signal acquisition system is shown in Supplementary Fig. 1. Four kinds of challenging scenarios were used in this experiment: lead vehicle autonomous emergency braking in a short distance, lead vehicle cut-in from left lane in a short distance, lead vehicle cut-in from right lane in a short distance and pedestrian crossing road from right scenarios. In this study, in order to make participants do not always keep stress, other scenarios were added in this study, other scenarios contain the scenario in which pedestrian does not cross road, the scenario in which vehicle does not cut in and so on. They are not relatively emergency scenarios for driving safety, compared with the above scenarios, therefore, the data in other scenarios were not analyzed.

The participants were requested to sit on a driving simulator and to look at the front scenario. Beside, the participants were required to press the keyboard when they hear a stimulating sound or they feel dangerous, they were not required to do other things. The motivation of adding stimulating sound is to judge whether or not the participants are focusing on those task by comparing the time delay in which participants pressed the keyboard when they hear a stimulating sound, and its detailed contents are shown in Supplementary Tables 2 and 3. The participants were asked to look at four segments in total in one day. When the participants completed a segment, the participants were asked to rest for 5 min, and it took 3 days for a participant complete this experiment.

**Figure 7:**
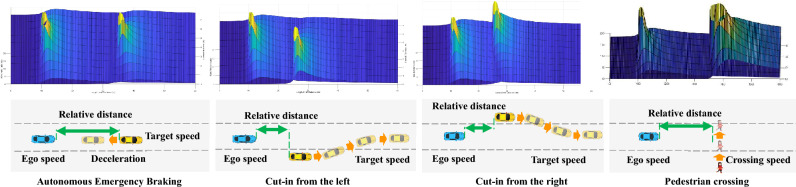
The sketch maps and risk fields at a moment of autonomous emergency braking in a short distance, vehicle cut in from the left lane, vehicle cut in from the right lane and pedestrian crossing road from the right scenarios.

In this paper, ego vehicle went straight on a three-lane road at 70 km/h, and the ego vehicle, a target vehicle and a pedestrian compose scenarios. For each participant, there were 288 scenarios, which were established based on VTD Software; there were 12 VTD segments, and the duration of each segment was approximately 13 min, each segment contained 24 scenarios and the scenario order was random, but there are no event before 1000 m in each VTD segment for making participant entry state. The detailed information about 288 scenarios is shown in Supplementary Tables 4 and 5. For twenty participants, the total sample numbers of lead vehicle autonomous emergency braking in a short distance, lead vehicle cut-in from left lane in a short distance, lead vehicle cut-in from right lane in a short distance and pedestrian crossing road from right are 280, 560, 500 and 660, respectively. But, thirteen autonomous emergency braking in a short distance sample, twenty-nine lead vehicle cut-in from left lane in a short distance samples, twenty lead vehicle cut-in from right lane in a short distance samples and 28 pedestrian crossing road from right samples were rejected owing to equipment failure. Those information is shown in Supplementary Table 6. The sketch maps and risk fields at a moment of four scenarios are shown in Fig 7.

## Data analysis process

The data analysis process included a data processing part and a data analysis part. Firstly, a stimulus moment was obtained based on the risk field, and the data of cerebral oxygen exchange was divided into low-risk and high-risk episodes; Secondly, low-risk features and high-risk features were extracted by K-SVD dictionary learning and mean values from low-risk and high-risk episodes, respectively; Finally, the T-value analysis method was used to assess the area sensitive to driving scenario risk in the prefrontal contex of passengers based on fNIRS for high-level automated vehicles, and GLM was used to analyze the quantification relationship between passengers’ mental activity and driving scenario risk. The data was dealt with by MATLAB 2020.b, python 3.11.3 and SPSS 25.0.

### Risk field

**Figure 8:**
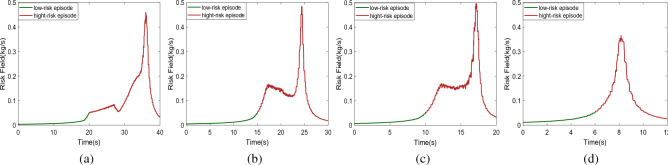
The risk field change cures in lead vehicle autonomous emergency braking in a short distance, lead vehicle cut-in from left lane in a short distance, lead vehicle cut-in from right lane in a short distance and pedestrian crossing road from right scenarios are shown in (**a**–**d**), the green curve and red curve indicate the risk in low-risk and high-risk episodes, respectively.

Risk field is a safety indicator, it may indicate a dangerous degree of driving scenario. In this study, the kinetic energy field^26^ was adopted to indicate driving scenario risk, the kinetic energy field is showed in Eq. (1), the kinetic energy field involves relative longitudinal distance, relative speed and other information, and those data may be obtained by Matlab/Simulink module.

1 $$\begin{aligned} {{\textbf {E}}}_{v}=\frac{GR_{2}M_{2}}{{\varvec{r}}^{k_{1}}}\frac{{\varvec{r}}^{k_{1}}}{\vert {\varvec{r}}^{k_{1}}\vert }e^{\left[ k_{2}v_{2}cos(\theta _{2})\right] } \end{aligned}$$

where $k_{1},k_{2}$ and *G* are three constants, $M_{2}$ indicates target vehicle mass, ${\varvec{r}}$ indicates the distance between target vehicle and ego vehicle, $v_{2}$ represents target vehicle speed, $R_{2}$ is road friction coefficient, and $\theta _{2}$ indicates the angle between ${\varvec{r}}$ and $v_{2}$. In application, $G=0.001$, $k_1=1$, $k_2=0.05$, $M=1705$, $R_2=1$, the episode in which ${{\textbf {E}}}_{v}>0.05$ is considered as a high-risk episode, and otherwise it is considered as a low-risk episode. Since those four scenarios were different, the times of low-risk episode and high-risk episode in autonomous emergency braking in a short distance scenario, vehicle cut in from the left scenario, vehicle cut in from the right scenario and pedestrian crossing road from the right scenario are 6 s, 15 s, 10 s, and 20 s. The risk field change cures of those four scenarios are shown in Fig. 8.

### fNIRS recording

$\triangle HbO$ and $\triangle HbR$ were collected using a blood oxygen monitoring device named as OctaMon+, which was provided by Artinis, a Dutch company. Those raw data were dealt with by NIRS-KIT^33^, which is a MATLAB toolbox for both resting-state and task fNIRS data analysis. The second-order polynomial detrending method was adopted to reduce the influence of data drift, motion artifacts were rectified by Temporal Derivative Distribution Repair^34^, and a band pass IIR filter (0.015 Hz to 0.08 Hz) was used to remove respiration, heart rate, blood pressure fluctuations, mayer waves noises, and others noises.Previous studies have shown that $\triangle COE$ is an effective index, which may indicate activity^26–28^, and it is showed in Eq. (2). In this study, the differences in $\triangle COE$ between the low-risk and high-risk episodes were analyzed.

2 $$\begin{aligned} \triangle COE=\frac{\triangle HbR -\triangle HbO }{\sqrt{2}} \end{aligned}$$

### Feature extraction

In this study, a K-SVD dictionary^29^ learning and mean value methods were used to extract features. K-SVD dictionary learning is a parametric low-dimensional representation learning method that can reconstruct high-dimensional input vectors in an unsupervised manner. The risk field data and cerebral oxygen exchange data were aligned by four events; the cerebral oxygen exchange data was divided into low-risk and high-risk episodes based on the risk field. The cerebral oxygen exchange data of all samples were extracted features by using K-SVD dictionary learning and mean value.

### Supplementary Information

Supplementary Information 1.

Supplementary Information 2.

## Data Availability

The data comes from Tsinghua Intelligent Vehicle Design and Safety Research Institute, it is publicly available as it has signed the “ethical statement” file. Please find the data and processing code via https://github.com/SOTIF-AVLab/fNIRS or please contact the corresponding author with any further queries regarding data availability.
