# Supplementary material for: Driving risk cognition of passengers in highly automated driving based on the prefrontal cortex activity via fNIRS
Source: Sci Rep. 2023 Sep 22;13:15839. doi: 10.1038/s41598-023-41549-9 (PMC10516872; doi:10.1038/s41598-023-41549-9)
Supplement: Supplementary file 1 — Supplementary Information 1. [file 41598_2023_41549_MOESM1_ESM.pdf]

Supplementary information

This supplementary information (SI) describes a human factor signal acquisition system to record data related to passenger status during driving simulation experiments. The SI also provides detail participant information, some methods which are used in our manuscript and detail scenario information. Beside, SI compares the results of those data which contain stimulating sounds and those data which do not contain stimulating sounds.
