# Supplementary material for: Driving risk cognition of passengers in highly automated driving based on the prefrontal cortex activity via fNIRS
Source: Sci Rep. 2023 Sep 22;13:15839. doi: 10.1038/s41598-023-41549-9 (PMC10516872; doi:10.1038/s41598-023-41549-9)
Supplement: Supplementary file 2 — Supplementary Information 2. [file 41598_2023_41549_MOESM2_ESM.pdf]

Driving risk cognition of passengers in highly automated driving based on the prefrontal cortex activity via fNIRS

Hong Wang[1,+], Xiaofei Zhang1[*,+], Jun Li[1] , Bowen Li[2], Xiaorong Gao[2,*], Zhenmao Hao[3], Junwen Fu[4], Ziyuan Zhou[1], and Mohamed Atia[4]

1. School of Vehicle and Mobility, Tsinghua University, Beijing, 100084, China
2. School of Medicine, Tsinghua University, Beijing, 100084, China
3. School of Computer Science, Carleton University, Ottawa ON, K1S5B6, Canada
4. Department of Systems and Computer Engineering, Carleton University, Ottawa ON, K1S5B6, Canada

* csezxf@126.com and gxr-dea@tsinghua.edu.cn

\+ these authors contributed equally to this work

Table of Contents

1 Supplementary Notes 1

1.1 Human factor signal acquisition system 1

1.2 Participants information 2

2 Supplementary methods 3

2.1 K-SVD dictionary 3

2.2 Statistical model 4

3 Supplementary Discussion 4

4 Description of Supplementary Data File 6

1 Supplementary Notes

1.1 Human factor signal acquisition system

This human factor signal acquisition system includes seven pieces of equipment, a lower computer, an upper computer, a driving simulator, a camera, audio, blood oxygen monitoring, and three screens. The structure of this human factor signal acquisition system is shown in Supplementary Fig 1. This lower computer is a Redhawk system, which is a real-time system, and runs Virtual Test Drive (VTD) software; the upper computer is a window system and runs Matlab/Simulink and Oxysoft software. The functions of Matlab/Simulink include the communication between the lower computer and upper computer by using User Datagram Protocol (UDP), the data parser of experiment scenarios which are built by VTD software, and the communication between the upper computer and Oxysoft software. Oxysoft software and blood oxygen monitoring device work together for acquiring the concentration changes of oxygenated hemoglobin and deoxygenated hemoglobin. The camera is used to record the status of the subject for the convenience of subsequent experimental data analysis.

This human factor signal acquisition system can be divided into three subsystems, image display subsystem, auditory stimulation subsystem, and passenger status recording subsystem. The image display subsystem includes three screens, they display experiment scenarios, passenger status which is recorded by the camera, and the brain signals of the passenger respectively. The auditory stimulation subsystem is audio that is controlled by an upper computer and can randomly make a prompt sound. The passenger status recording subsystem consists of a camera, blood oxygen monitoring device, and keyboard, it is the most important part, and it can record the brain signals of passengers which are inspired by experiment scenarios. The auditory stimulation subsystem and the passenger status recording subsystem work together, when the auditory stimulation subsystem emits a prompt sound, subjects need to press the keyboard as the feedback of hearing this sound, and the purpose is to keep participants' attention highly concentrated. If participants do not tap the keyboard after hearing the sound, the corresponding data in this time will be rejected in the subsequent experimental data analysis phase.

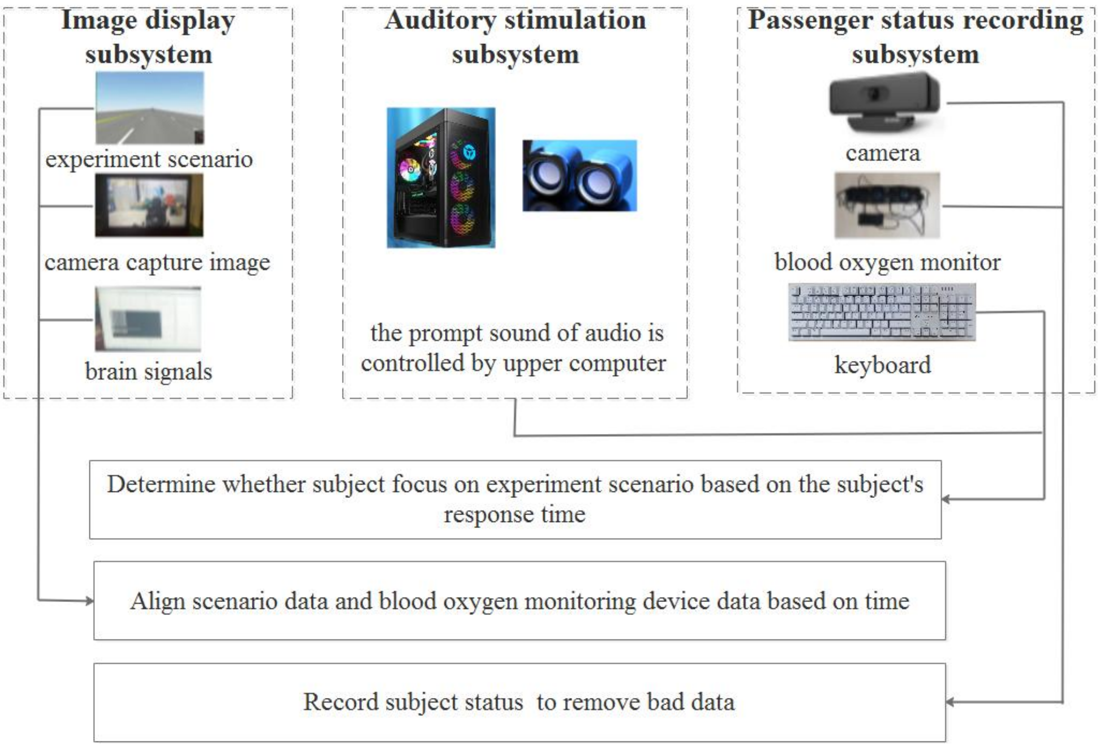

Supplementary Figure 1: the structure of the human factor signal acquisition system

1.2 Participants information

A total of 20 participants completed this driving simulator experiment, and the participant information is shown in Supplementary Table 1.

Supplementary Table 1 Subject information

NO.	Sex	Age	Driving experience
1	Male	24	0
2	Female	46	0
3	Female	42	3
4	Male	41	17
5	Male	25	7
6	Male	21	0
7	Male	32	0
8	Male	22	0
9	Male	28	0

10	Male	24	0
11	Female	25	0
12	Male	25	0
13	Female	25	0
14	Female	32	0
15	Male	31	0
16	Male	24	0
17	Male	41	10
18	Male	33	3
19	Male	24	1
20	Male	26	5

2 Supplementary methods

2.1 K-SVD dictionary

$X = [x_1, ..., x_n] \in \mathbb{R}^{m\times n}$ is used to represent a matrix that contains n data samples $x_i \in \mathbb{R}^m$. Through the dictionary learning model and sparse representation model, we decompose high-dimensional data into

$$x_i = D\alpha_i \quad (1)$$

where $D = [d_1, \cdots, d_k]$, and $i = 1, ..., n$.

Here is the dictionary $D \in \mathbb{R}^{m\times k}$ is the public dictionary of the data point x_i, and α_i is its corresponding sparse representation. We assume that all columns of the dictionary D have a unit norm, that is, $\|d_j\|_2 = 1$, for all $j = 1, ..., k$. Solving sparse decomposition is a well-known dictionary learning problem, the common method of the dictionary learning model is an optimization problem, so we construct the following formula through the elastic net algorithm,

$$\min_{D,\alpha} H(D, A) \quad \text{s.t } D \in \mathbb{R}(m, k) \quad (2)$$

$$H(D, A) := \frac{1}{2}\|X - DA\|_2^2 + \sum_{i=1}^{n}\left\{\lambda_1\|\alpha_i\|_1 + \frac{\lambda_2}{2}\|\alpha_i\|_2\right\} \quad (3)$$

where $A = [\alpha_1, \cdots, \alpha_n]$, λ_1 and λ_2 are two constants.

2.2 Statistical model

- **T-test**

T-test is a statistical model, the p-value and t-value are two values of the T-test, and the t-value is a ratio between the difference between two groups and the difference within the groups. A larger t-value indicates more differences between groups, smaller t-values indicate more similarities between groups; the p-value is the probability, when it is smaller than 0.1, the t-test result means that the test rejects the null hypothesis at the 10% significance level, and there are significant differences.

- **Wilcoxon Signed Rank Test**

Wilcoxon Signed Rank Test is a nonparametric test. Two data samples are matched if they come from repeated observations. Wilcoxon Signed Rank Test is used to determine whether the overall distribution of the corresponding data is the same without assuming that the data obey a normal distribution. It gets the test statistic Z value, the Z value reflects the different degrees of matching data, and the larger the Z value, the greater the difference between groups.

- **Generalized Linear Model**

In statistics, a generalized linear model (GLM)[1] is a generalization of the ordinary linear regression model, its response variable needs to meet an error distribution model, and this distribution model only needs to satisfy normal distribution. GLM consists of three parts: random component, systematic component, and link function. The random component specifies the probability distribution of the response variable; the systematic component specifies the explanatory variables in the model; more specifically, the link function specifies the link between the random and the systematic components.

3 Supplementary Discussion

In this experiment, the participants were required to press the keyboard when they hear a stimulating sound or they feel dangerous. The motive of adding stimulating sound is to judge whether or not the participants are focusing on those tasks by comparing the time delay in

[1] https://online.stat.psu.edu/stat504/lesson/6/6.1

which participants pressed the keyboard when they hear a stimulating sound. We compared the results of those data which contain stimulating sounds and those data which do not contain stimulating sounds in Supplementary Information. The detailed results are shown in Supplementary Table 3 and Supplementary Table 4. Although the T-values based on mean value features between low-risk and high-risk episodes about those two kinds of data are different, the T-values about those two kinds of data in channel 8 are maximum. This result indicates that the conclusions of those two kinds of data are consistent, and the mental activities of passengers caused by driving scenario risk in the Brodmann area 10 are very active.

Supplementary Table 2: The results of those two kinds of data

Result	Scenario	Ch1	Ch2	Ch3	Ch4	Ch5	Ch6	Ch7	Ch8
The result of those data which contain stimulating sounds	SAEB	-1.284	-0.817	-0.181	0.745	-0.844	-0.145	0.426	**1.199**
	LCI	-1.181	1.610	1.529	1.884	0.797	0.998	2.690	**3.205**
	RCI	-1.120	1.287	0.451	1.907	-1.002	0.888	1.102	**2.572**
	RPCR	1.139	2.343	3.052	4.043	1.075	3.423	1.755	**4.439**
The results of those data which do not contain stimulating sounds	SAEB	-1.074	-0.661	0.173	0.891	-0.958	-0.256	0.387	**1.418**
	LCI	-0.739	1.718	1.419	1.961	0.648	1.436	1.772	**2.396**
	RCI	-0.724	1.277	0.413	2.040	-0.711	1.768	0.795	**2.494**
	RPCR	1.649	2.617	3.584	4.630	1.543	3.432	2.065	**4.648**

1. Ch1 stands for the channel i

Supplementary Table 3:The sample numbers of those two kindss of data

Scenario	The sample numbers of those data which do not contain stimulating sounds	The sample numbers of those data which contain stimulating sounds
SAEB	159	267
LCI	352	531
RCI	396	480
RPCR	603	632

4 Description of Supplementary Data File

In this paper, scenarios are established based on VTD software, there are 12 VTD segments, the duration of each segment is approximately 13 minutes, and each segment contains 24 scenarios. There are total of 288 scenarios. Those scenarios belong to fourteen kinds, and The video (section 1-section6) of those fourteen kinds of scenarios is uploaded to GitHub: https://github.com/SOTIF-AVLab/fNIRS. The order of those fourteen kind scenarios are lead vehicle cut out to right lane, lead vehicle cut out to left lane, lead vehicle autonomous emergency braking in a long distance, lead vehicle cut-in from left lane in a short distance, lead vehicle cut-in from right lane in a short distance, lead vehicle cut-in from right lane in a long distance, lead vehicle autonomous emergency braking in a short distance, lead vehicle cut-in from left lane in a long distance, lead vehicle run on the right lane and do not cut-in, pedestrian crossing road from right, pedestrian crossing road from left, pedestrian standing on the left side and do not cross the road, pedestrian standing on the right side and do not cross the road, lead vehicle run on left land and do not cut-in. Ego vehicle runs straight at 70km/h, and its corresponding scenario descriptions are shown in Supplementary Table 4, and the number of those fourteen kinds of scenarios in 12 VTD segments are shown in Supplementary Table 5.

Supplementary Table 4: the information of the fourteen kinds of scenarios

No	Scenario name	Scenario Description
1	lead vehicle cut out to the right lane	The lead vehicle runs at 50km/h. When the distance between the lead vehicle and the ego vehicle is 50m, the lead vehicle cutes out to the right lane
2	lead vehicle cut out to the left lane	The lead vehicle runs at 40km/h. When the distance between the lead vehicle and the ego vehicle is 25m, the lead vehicle cuts out to the left lane
3	lead vehicle autonomous	The lead vehicle runs at 50km/h. When the

	emergency braking in a long distance	distance between the lead vehicle and the ego vehicle is 35m, the lead vehicle brake to 20km/h; When the distance between the lead vehicle and ego vehicle is 19m, the lead vehicle cuts out
4	lead vehicle cut in from the left lane in a short distance	The lead vehicle runs at 40km/h. When the distance between the lead vehicle and the ego vehicle is 25m, the lead vehicle cut-in from the left lane
5	lead vehicle cut in from the right lane in a short distance	The lead vehicle runs at 40km/h. When the distance between the lead vehicle and the ego vehicle is 35m, the lead vehicle cut-in from the right lane
6	lead vehicle cut in from the right lane in a long distance	The lead vehicle runs at 40km/h. When the distance between the lead vehicle and the ego vehicle is 60m, the lead vehicle cut-in from the right lane
7	lead vehicle autonomous emergency braking in a short distance	The lead vehicle runs at 55km/h. When the distance between the lead vehicle and the ego vehicle is 40m, the lead vehicle brake to 10km/h; When the distance between the lead vehicle and the ego vehicle is 18m, the lead vehicle cuts out
8	lead vehicle cut in from the left lane in a long distance	The lead vehicle runs at 40km/h. When the distance between the lead vehicle and the ego vehicle is 60m, the lead vehicle cut-in from the left lane
9	lead vehicles run on the right land and do not cut-in	The lead vehicle runs at 40km/h on the right land and does not cut-in

10	pedestrian crossing the road from right	When the distance between the lead vehicle and the pedestrian is 55m, the pedestrian crosses the road from the right at 10.91km/h
11	pedestrian crossing the road from left	When the distance between the lead vehicle and the pedestrian is 120m, the pedestrian crosses the road from the left at 10.91km/h
12	a pedestrian standing on the left side and does not cross the road	Pedestrian stand on the left side and do not cross the road
13	a pedestrian standing on the right side and does not cross the road	Pedestrian stand on the right side and do not cross the road
14	lead vehicle run on left land and do not cut-in	The lead vehicle runs at 40km/h on left land and does not cut-in

Supplementary Table 5: the number of the fourteen kinds of scenarios

No	Scenario name	Number
1	lead vehicle cut out to the right lane	9
2	lead vehicle cut out to the left lane	14
3	lead vehicle autonomous emergency braking in a long distance	13
4	lead vehicle cut in from the left lane in a short distance	28
5	lead vehicle cut in from the right lane in a short distance	25
6	lead vehicle cut in from the right lane in a long distance	22
7	lead vehicle autonomous emergency braking in a short distance	14
8	lead vehicle cut in from the left lane in a long distance	19
9	lead vehicle run on the right land and do not cut-in	19
10	pedestrian crossing the road from right	33
11	pedestrian crossing the road from left	31
12	a pedestrian standing on the left side and does not cross the road	19
13	Pedestrians standing on the right side and do not cross the road	25

14	lead vehicle run on left land and do not cut-in	17

In this paper, lead vehicle autonomous emergency braking in a short distance, lead vehicle cut-in from the right lane in a short distance and lead vehicle cut-in from the left lane in a short distance, and pedestrian crossing the road from the right scenarios are considered for analyzing the mental activities of passengers caused by driving scenario risk. Twenty participants completed this experiment. Some data were rejected, owing to equipment reasons. 1910 groups were finally obtained, due to there is difference between those four scenarios, four different window time are adopted, and detailed information is shown in supplementary table 6

Supplementary Table 6: the detailed information about the samples

No	Scenario	Data number	Data dimension	Window time
1	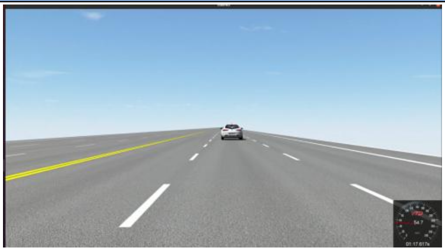 lead vehicle autonomous emergency braking in a short distance	267	（268,8,4000）	20
2	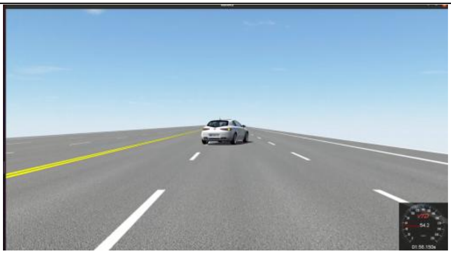 lead vehicle cut in from the left lane in a short distance	531	（535,8,3000）	15
3	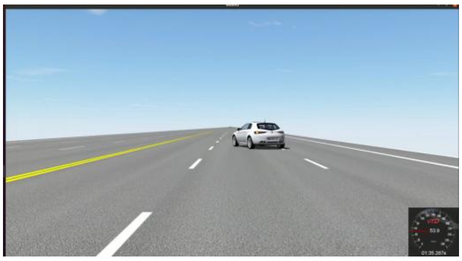	480	（482,8,2000）	10

	lead vehicle cut in from the right lane in a short distance			
4	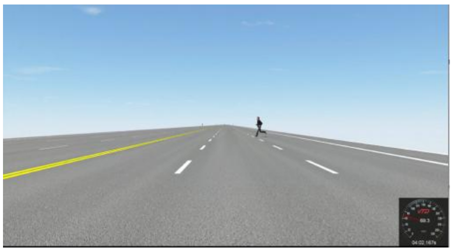 pedestrian crossing the road from right	632	（634,8,1200）	6
